# Supplementary material for: Genomic Instability Is Defined by Specific Tumor Microenvironment in Ovarian Cancer: A Subgroup Analysis of AGO OVAR 12 Trial
Source: Cancers (Basel). 2022 Feb 25;14(5):1189. doi: 10.3390/cancers14051189 (PMC8909387; doi:10.3390/cancers14051189)
Supplement: Supplementary file 1 [file cancers-14-01189-s001.zip › Supp Figure S1-S3.pdf]

PFS according to treatment group in whole population

A

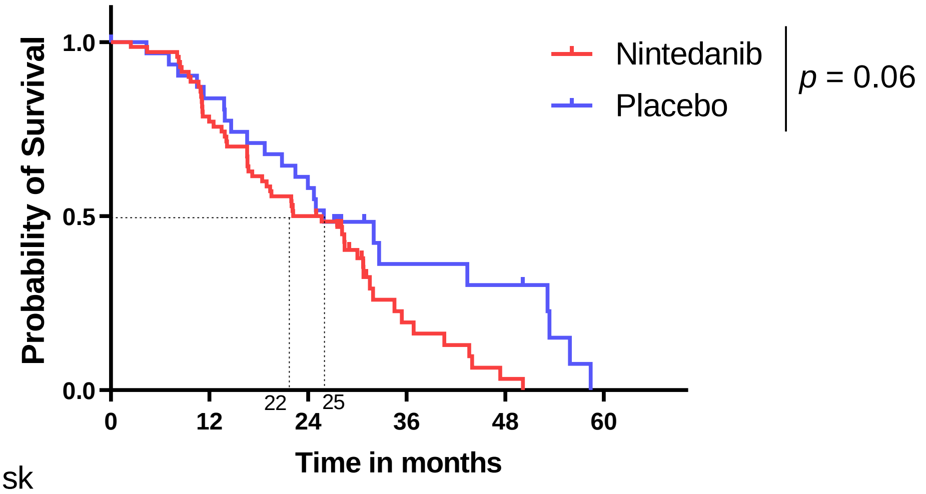

Number at risk

Nintedanib	70	54	34	6	1	0
Placebo	33	26	18	6	5	0

B

OS according to treatment group in whole population

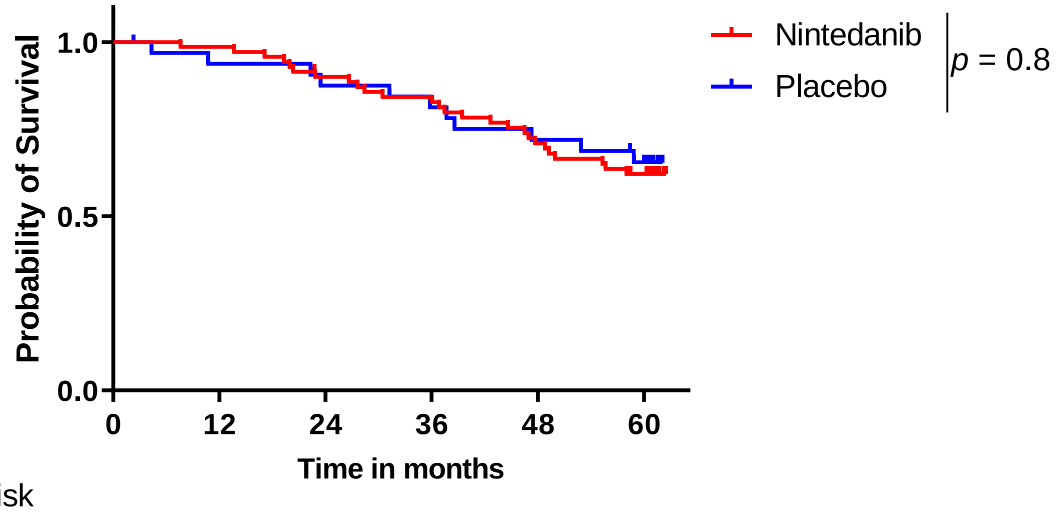

Number at risk

Nintedanib	70	69	62	58	48	41
Placebo	33	30	28	26	23	19

Figure S1. (A) PFS according to treatment group in whole population; (B) OS according to treatment group in whole population

PFS

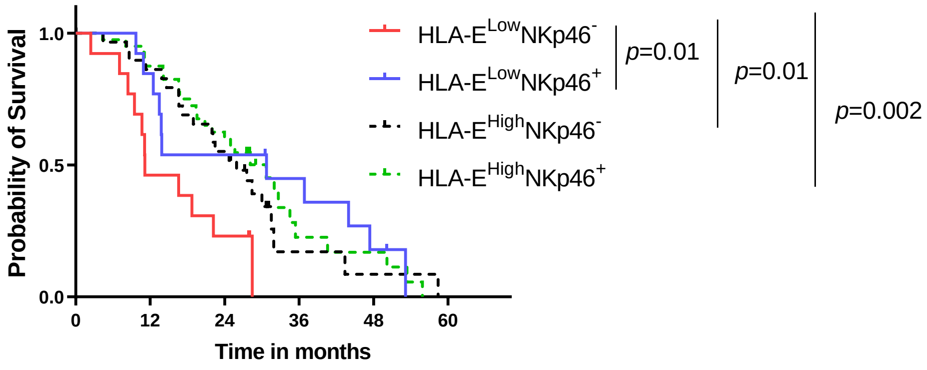

0S

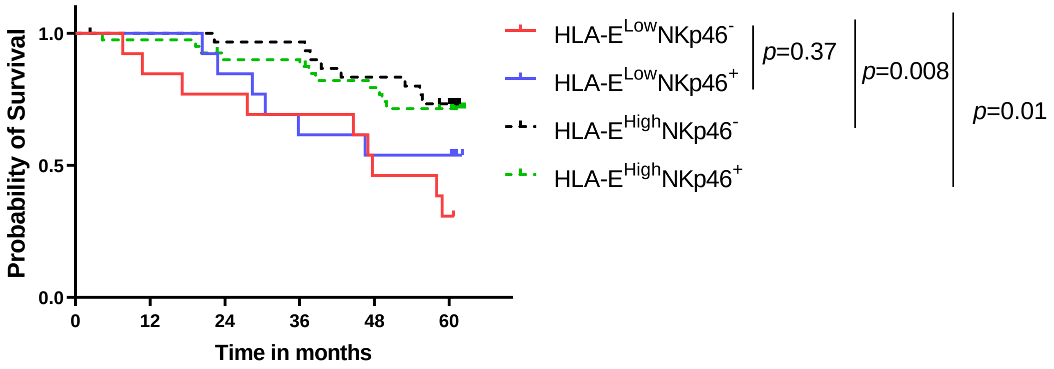

Figure S2. PFS and OS according HLA-E and NKp46 expression

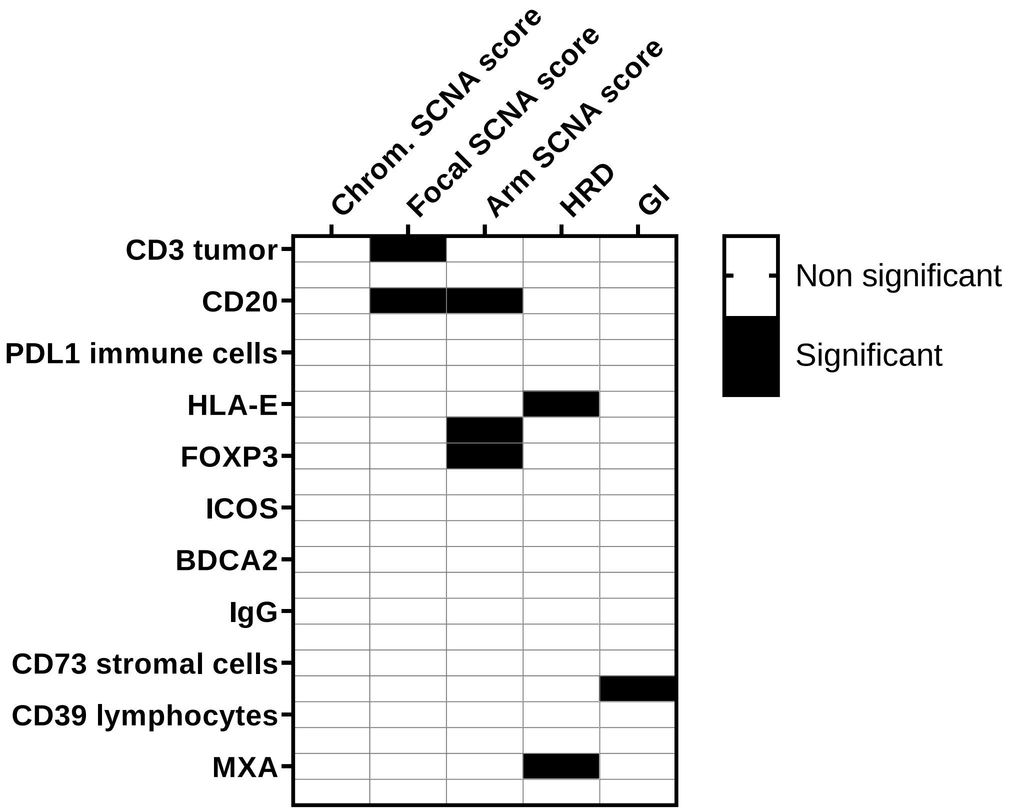

Figure S3. Heatmap of relationship between genomic signatures and immune parameters
